# Centering Pregnancy in Missouri: A System Level Analysis

**DOI:** 10.1155/2014/285386

**Published:** 2014-02-13

**Authors:** Pamela K. Xaverius, Mary Alice Grady

**Affiliations:** ^1^Saint Louis University, College for Public Health and Social Justice, 3545 Lafayette Avenue, Salus Center, Room 474, Saint Louis, MO 63104, USA; ^2^Centering Healthcare Institute, Boston, MA 02111, USA

## Abstract

*Background*. Centering Pregnancy (CP) is an effective method of delivering prenatal care, yet providers have been slow to adopt the CP model. Our main hypothesis is that a site's adoption of CP is contingent upon knowledge of the CP, characteristics health care personnel, anticipated patient impact, and system readiness. *Methods*. Using a matched, pretest-posttest, observational design, 223 people completed pretest and posttest surveys. Our analysis included the effect of the seminar on the groups' knowledge of CP essential elements, barriers to prenatal care, and perceived value of CP to the patients and to the system of care. *Results*. Before the CP Seminar only 34% of respondents were aware of the model, while knowledge significantly after the Seminar. The three greatest improvements were in understanding that the group is conducted in a circle, the health assessment occurs in the group space, and a facilitative leadership style is used. Child care, transportation, and language issues were the top three barriers. The greatest improvements reported for patients included improvements in timeliness, patient-centeredness and efficiency, although readiness for adoption was influenced by costs, resources, and expertise. *Discussion*. Readiness to adopt CP will require support for the start-up and sustainability of this model.

## 1. Introduction

Prenatal care is an important public health priority and often the first line of defense in preventing poor birth outcomes [1]. Unfortunately, traditional prenatal care has been unsuccessful at improving birth outcomes, and in fact, when a larger quantity of care is provided over a longer period of time, birth outcomes have not improved [2, 3]. If the US is to improve patient outcomes through high quality care, we must engage in serious and sustained efforts directed at providers, patients, and the systems in which they work [4]. The Institute of Medicine (IOM) recommends that, for clinical care to be of a high quality, care must also value the patients [5], measurable by the six dimensions of safety, effectiveness, patient-centeredness, timeliness, efficiency, and equitableness of care [6]. Further, patient-centered prenatal care should include advice and counseling on nutrition, vitamin use, smoking, alcohol and drug use cessation, breastfeeding, and maternal weight gain [7]. Substantially improving birth outcomes requires a paradigm shift from the more traditional prenatal care to a more comprehensive mode of prenatal care delivery [8].

Health care systems strive to give quality care but are often deterred by poorly designed systems that are resistant to change [5, 9]. The gap between research and practice can be exacerbated by inadequate infrastructure and systems organization to support the translation of innovations into practices [5]. Thus, moving efficacious interventions from research settings to programs already in practice is very challenging [10, 11]. Indeed interventions found efficacious in controlled settings, often only appeal to the most motivated participants in real-world settings, and are not easily adopted [12]. Factors associated with adoption include political and cultural fit, cost, level of resources/expertise required, and ease of adoption [13, 14]. What stands in the way of adoption includes insufficient information regarding the innovation by those who could implement the innovation, coupled with lack of education, resources, and leadership commitment to making a change [7].

One such innovation in prenatal care is Centering Pregnancy (CP), a group model of prenatal care, with a reduced schedule of visits which includes assessment, education, and support in the clinical space [15, 16]. The model also adheres to the IOM's recommendations for tailoring provider services to patient needs [5]. In one pilot study of 111 women who received CP, 91% were satisfied with the assessment occurring in the clinical space, 94% learned a lot about prenatal care, and 98% enjoyed the support of the group [17]. Another study found 96% of the women preferred receiving their prenatal care in groups, and providers were very satisfied with the efficiency of care [18]. The combination of satisfaction, good outcomes, and effective delivery of care has led to further evaluation of the model [19]. An evaluation of prenatal care at a site in St. Louis reported preterm birth rates at 23.2% and 25.7% before CP was implemented (1998 and 2001, resp.) and 10.5% after CP was implemented [20]. Another prospective, matched cohort evaluation of CP that included 458 pregnant women who began prenatal care at 24 or less weeks of gestation reported that CP resulted in significantly higher birth weight infants [21]. In a large randomized controlled trial, which focused on 14–25-year old pregnant women (*n* = 1,047), the risk for preterm birth was significantly reduced by 33% for women in group care, with the effects strengthened for African-American women, whose risk was reduced by 41% [22]. In a recent cohort study (*n* = 4,083), significant reductions in racial disparities were also found [23]. Additionally, women who received CP were more likely to receive optimal prenatal care and initiate breast feeding at a higher rate, while the enhanced care model costs no more than standard prenatal care or delivery. This groundswell of research shows that CP improvements in birth outcomes could have a huge public health impact if widely adopted, which is in stark contrast to the lack of improved outcomes seen by investing in an increased number of prenatal care visits alone [3].

If CP is to become the evidence-based standard to deliver prenatal care, it will be essential to change the system in which usual prenatal care operates [24]. RE-AIM is a model that evaluates the translation of evidence-based interventions into systems on five dimensions: reach, effectiveness, adoption, implementation, and maintenance. Reach, effectiveness, and implementation are consumer based measures that refer to the ability of the implemented innovation to reach the target population (i.e., pregnant women) and to improve their birth outcomes. Adoption and implementation are system level assessments that consider the uptake and consistent delivery of the innovation and maintenance measures the sustainability of the innovation over time. Collectively, these five dimensions provide necessary information to shift our current system of prenatal care towards an improved system of care. Since the original RE-AIM paper in 1999, there have been approximately 100 publications on RE-AIM by a variety of authors in fields as diverse as aging [25], cancer screening [26], dietary change [27], physical activity [28], health policy [29], environmental change [30], chronic illness self-management [31], well-child care [32], eHealth [33], worksite health promotion [34], women's health [35], smoking cessation [36], and practice-based research [37]. The RE-AIM framework provides valuable framework for evaluating the translation of health care innovations such as CP into clinical settings [38, 39].

This paper evaluates the findings from a project designed to translate CP into healthcare clinics in Missouri and examines the relationship between educating providers on this innovative model of prenatal care and the subsequent uptake of the CP model. We also evaluate the system level impact (i.e., reach, effectiveness, adoption, implementation, and maintenance) and patient level impact (i.e., safety, effectiveness, patient-centeredness, timeliness, efficiency, and equitableness of care), as it relates to differences between adopters and nonadopters of the CP model. These findings will have important implications for translating CP into prenatal care practice.

## 2. Methods

Using a matched, pretest posttest observational design, we assessed health care personnel's knowledge about CP before and after a one-hour informational seminar.

### 2.1. Participants

Potential prenatal care providers were identified from the list of federally qualified health care centers located on the Midwest Primary Care Association's website and through recommendations by providers and colleagues of the primary author. A final list of 40 prenatal care sites or prenatal care essential service providers was created, and 17 of those sites held informational seminars at their sites between 2008 and 2011. Approximately 245 people attended those seminars, of which 223 completed pretest and posttest surveys were collected, for a response rate of 91.0%.

### 2.2. Independent Variable

An hour-long information session was delivered to all participants. Informational seminars provided education on the epidemiology of poor birth outcomes in Missouri, a review of the evidence for improved birth outcomes for women exposed to the CP model of prenatal care, high levels of patient satisfaction with CP, and an overview of the essential elements of CP necessary to be an approved CP model of care. The sessions were jointly delivered by an epidemiologist and a certified nurse midwife. A light meal or snack was provided for all people in attendance. Sites interested in adopting CP within their clinics were offered the opportunity to attend a follow-up two-day training session on the delivery of CP and consultation and support during mode implementation and through their first few group sessions.

### 2.3. Survey

The survey was developed to assess demographic characteristics of individual respondents, characteristics of prenatal care practices, knowledge of the essential elements of the CP model of prenatal care, and strengths and barriers of prenatal care. Pre- and posttest surveys were stapled together and distributed to all participants. Respondents were instructed to complete the pretest survey before the start of the presentation. After the presentation, respondents were instructed to complete a posttest survey. Surveys were collected at the conclusion of the information session.

Demographic variables included sex, race, ethnicity, age category (18–24, 25–39, 40–55, and 55 years or older), and job characteristics (“do you provide prenatal care,” “what is your profession (physician, nurse practitioner, certified nurse midwife, physician assistant, and other),” and “which of the following categories represents the majority of your current professional activity? (patient care, research, teaching, administration, and other)”), average weekly pregnant patient volume at all office locations, average minutes spent with each pregnant patient, and work location (“urban,” “rural”).

Essential elements of CP included patient self-assessment, provider in group space, education and support, formal and informal socialization, group cohesion, patient and provider consistency, facilitative leadership style, session plans, core content, group guidelines, conducting the session in a circle, optimal group size, family support options, and ongoing evaluation. Respondents were also asked if they had ever heard of CP and if they thought there was increased value in providing CP prenatal care. If they had heard of CP before the seminar, respondents were asked to identify essential elements on both the pretest and posttests. If they had not heard about CP before the seminar, respondents were asked to identify essential elements on the posttest only.

Prenatal care practice was measured by patient-level and system-level measures. Patient-level measures included safety (i.e., avoiding injury for those we intend to help), effectiveness (i.e., providing services based on scientific knowledge to all those likely to benefit), patient centeredness (i.e., care that is respectful and responsive to individual preferences, needs, and values; letting these values guide clinical decisions), timeliness (i.e., care that reduces wait and harmful delays for provider and recipient), efficiency (i.e., care that avoids waste of supplies, equipment, ideas, and energy), and equitableness (care that does not vary in quality because of personal characteristics such as gender, race, geography, and socioeconomic status). Two responses were recorded regarding this indicator, one regarding usual care and one regarding switching to CP care. Responses regarding usual care were based on a 5-point Likert scale and recoded as “low” if respondents selected “not at all,” “slight,” or “average” and recoded as “high” if the respondents selected “strong” or “very strong.” Responses regarding switching to CP care were based on a three-point scale and recoded to a dichotomous scale of “improve” if the respondents selected “improve” and “not improve” if the respondents selected “stay the same” or “worsen.” System level measured was based upon RE-AIM variables and was coded as “low,” “medium,” or “high” on the following dimensions: reach (participation of women attending prenatal care), efficacy (impact on birth outcomes), adoption (cost, resources, and expertise), implementation (fidelity of quality of prenatal care), and maintenance (sustained model of prenatal care).

Finally, respondents were asked if the following were “strengths,” “barriers,” or “uncertain” in their relationship to prenatal care practices: administrative support, nursing support, physician support, professional group leadership skills, client base, current client satisfaction, patient flow, other prenatal education programs, language issues, child care, transportation, and parking.

### 2.4. Analysis

Analysis focused on differences in understanding the essential elements, prenatal care practice, before and after an information session, and barriers to switching to CP care. The analysis of prenatal care practice measures (i.e., patient level and system level) was restricted to those respondents that worked for a direct service site and compared. Mean scores are presented for all variables. chi-square tests were used to test for significant differences between groups (adopters and nonadopters), for dichotomous and categorical variables, with no more than 20% of the cells having less than five, meeting the assumptions of chi-square analysis. McNemar's chi-square (binomial test) test was used for paired categorical data. *t*-tests were used to measure significance of differences between pretest and posttest and between groups (adopters and nonadopters) regarding continuous variables.

## 3. Results

### 3.1. Respondent and Site Characteristics

Two-hundred and twenty-three respondents completed pre- and posttest surveys. The majority of respondents were female (80.5%), white, nonhispanic (70.6%), and between the ages of 25 and 39 years (44.4%). The majority of respondents provided care for pregnant women (76.1%). Thirty-four percent of participants heard of CP before the information session, and after the information session, 91.2% understood the increased value of CP and 91.7% could provide a brief explanation of CP. CP seminars were delivered to 17 different sites, with an average patient volume of 55 patients a week and an average patient visit lasting 18 minutes. Of the 17 sites, 15 provided direct clinical prenatal care and thus were eligible to adopt CP. Of the 15 sites, eight sites decided to adopt CP as their model of prenatal care (adopters) and seven sites did not (nonadopters). Characteristics of adopters were significantly different than nonadopters, in terms of age (*χ* ^2^[3] = 20.1; *P* = 0.000), race (*χ* ^2^[3] = 27.3; *P* = 0.000), providing care to pregnant women (*χ* ^2^[1] = 13.8; *P* = 0.000), and having heard of CP (*χ* ^2^[1] = 24.188, *P* = 0.000). There was no statistically significant difference between adopters and nonadopters in terms of weekly patient volume or time spent with each patient. See Table 1.

### 3.2. CP Essential Elements

Out of the sample of 223, 206 people answered the question “had you heard of Centering Pregnancy?” with 34% (*n* = 70) reporting “yes.” Out of the sample of 223, 171 participants answered the posttest assessment of the essential elements and the correct average percent was 81.4% (see Table 2). Of those who had heard of CP before the information session (*n* = 70), a paired samples *t*-test revealed a statistically significant posttest improvement in the overall understanding of the 14 essential elements of CP (pretest average = 63.5% and posttest average = 88.4%) (*t* = − 7.052, *P* = 0.001), with significant improvements found in 11 of the 14 essential elements.

**Figure 1:**
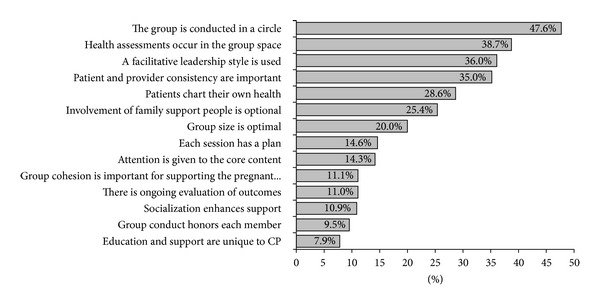
Learning curve: average percent improvement between time 1 and time 2 regarding essential elements.

Secondary analysis found significant improvements in 11 of the 14 items tested regarding essential elements, with the top five improvements included (1) the group is conducted in a circle (47.6%; *t* = − 7.508, *P* = 0.000), (2) the health assessment occurs in the group space (38.7%; *t* = −5.231, *P* = 0.000), (3) a facilitative leadership style is used (36.0%; *t* = −5.133, *P* = 0.000), (4) patient and provider consistency are important (35.0%; *t* = − 4.475, *P* = 0.000), and (5) patients chart their own health (28.6%; *t* = − 4.349, *P* = 0.000) (Figure 1).

### 3.3. Prenatal Care Practice: Patient Levels and System Levels

Overall, the highest proportion of respondents reported that if they were to change from usual care to CP care, equitableness (49%), effectiveness (64.2%), efficiency (72.3%), patient-centeredness (74.3%), and timeliness (77.0%) would improve.  Table 3 reports the findings overall and is stratified by adopter/nonadopter status. Overall, when matching posttest responses to whether these patient-level indicators would improve with CP care, with pretest identification of usual care practices as high or low on the same patient-level dimensions, significantly different proportions were found for equitable (46.5% versus 65.0%, *P* > 0.001), effectiveness (61.5% versus 65.0%, *P* > 0.001), efficiency (66.7% versus 79.7%, *P* > 0.001), patient-centeredness (73.2% versus 77.8, *P* > 0.001), and timeliness (73.3% versus 80.8%, *P* > 0.001) but not for safety (32.4% versus 43.2%, *P* = 0.063). Between adopters and nonadopters, a larger proportion of adopters reported that five of the six patient-level indicators would improve (equitableness: 52.1% versus 43.4%; effectiveness: 66.7% versus 59.6%; efficiency: 76.8% versus 64.2%; patient-centeredness: 80.2% versus 63.6%; and timeliness: 77.1% versus 76.9%, resp.) if they switched to CP but not regarding safety (34.0% versus 37.3%, resp.). Results were similar among the subset of adopters, with significantly different proportions between high and low usual care groups on equitableness (49.4% versus 66.7%, *P* > 0.001), effectiveness (63.3% versus 82.4%, *P* = 0.001), efficiency (73.1% versus 81.4%, *P* = 0.001), patient centeredness (80.6% versus 79.2%, *P* = 0.001), and timeliness (70.8 versus 83.3%, *P* = 0.001) but not safety (29.6% versus 46.2%, *P* = 0.311). Among nonadopters, significantly different proportions were found between high and low usual care groups regarding efficiency (56.3% versus 76.2%, *P* > 0.01) and timeliness (77.8% versus 76.0%, *P* > 0.006), but cell size was too small to calculate the McNemar test for the other patient-level indicators. The top three improvements, overall, were reported first regarding timeliness (77.0%), second for patient-centeredness (74.3%), and third for efficiency (72.3%), with the greatest proportion of respondents among adopters identifying patient-centeredness to improve (80.2%).

System level measures focused on RE-AIM constructs. Current prenatal care practices were rated according to the RE-AIM dimensions; 16.0% rated reach as low; 13.1% rated efficacy as low; 21.2% rated adoption as low; 7.1% rated implementation as low; and 8.2% rated maintenance as low. See Table 4. In contrast, According to opinions on CP, 1.8% rated reach as low, 0.6% rated efficacy as low, 11.5% rated adoption as low, 1.3% rated implementation as low, and 4.4% rated maintenance as low. chi-square analysis revealed a significant difference with adoption (*χ* ^2^[4] = 13.045, *P* < 0.011) and maintenance (*χ* ^2^[4] = 11.197, *P* < 0.024), with current prenatal care practices rating adoption as high 12.9% of the time and CP rating Adoption as high 42.3% of the time.

### 3.4. Strengths and Barriers

The top three strengths of current prenatal care services were physician support (82.8%); nurse support (79.7%); and client base (62.8%). The top three strengths seen for CP included professional leadership skills (65.1%); prenatal care education (59.5%); and client base (57.5%). The top three barriers were the same for current prenatal care and CP, that is, child care (44.3% and 42.0%, resp.); transportation (41.4% and 39.1%, resp.); and language issues (29.9% and 28.1%, resp.) (see Table 5). When comparing differences between current prenatal care and CP care, significant differences were found regarding administrative support (*χ* ^2^[4] = 38.68, *P* < 0.000); language issues (*χ* ^2^[4] = 51.39, *P* < 0.000); child care issues (*χ* ^2^[4] = 33.85, *P* < 0.000); and transportation (*χ* ^2^[4] = 41.45, *P* < 0.000). No significant differences were found between adopters and nonadopters on strengths/barriers of current prenatal care practices; however, significant differences were found if their practice switched to CP regarding language issues (*χ* ^2^ = 6.343, df = 2, *P* = 0.042). While 29.5% of adopters saw language issues as a strength, 51.9% of the nonadopters saw language issues as a strength.

## 4. Discussion

Health care personnel knew very little about the CP model of care (only 34%), although after a brief information session, 91.2% of the respondents reported that they understood the increased value of CP (i.e., group prenatal care) and 91.7% reported that they could give a brief explanation of the content and goals of the CP model of care. Furthermore, these brief CP seminars were effective at translating the essential elements as an important step towards a site's readiness to implement CP. The greatest improvements in knowledge were that the group is conducted in a circle (47.6% to 95.2%); health assessment occurs in the group space (32.3% to 71.0%); and facilitative leadership style is used (31.2% to 67.2%). A site's readiness to implement CP will require having the space to conduct group appointments and having staff trained in brief assessments and a facilitative leadership style.

There are both strengths and barriers to shifting to a new model of care. The top three strengths seen for CP included professional leadership skills (65.1%); another prenatal care education (59.5%); and client base (57.5%). The top three barriers were the same for current prenatal care and CP, child care (44.3% and 42.0%); transportation (41.4% and 39.1%); and language issues (29.9% and 28.1%), respectively. Implementation of the CP model shifts physician and nurse activities to more prenatal education and leadership activities. However, support and resources for physicians and nurses may assist care givers in this model. Barriers remained constant, having more to do with patient resources than with type of prenatal care.

The greatest improvements anticipated for patients included improvements in timeliness (77.0%), patient centeredness (74.3%), and efficiency (72.3%). A significant impact on the system's transition to CP was also found regarding a system's readiness to adopt (i.e., costs, resources, and expertise) and sustain this model of care. Sites that eventually adopted CP had a significantly higher prevalence of providers at the CP seminar who were younger and White, provided care to pregnant women, and had previously heard of CP. This population of health care personnel appears to be the early adopters of CP, setting the stage for increased knowledge of CP for more heterogeneous populations of health care providers.

There are a number of limitations to this study. First, this data is based upon self-report and is only representative of those people who attended CP seminar. Differences were not found regarding patient characteristics for nonadopters, which may be due to the small sample size within this subset. Differences in RE-AIM dimensions were asked, without indicating directionality. Thus, while differences were reported to be high, it is possible that respondents considered that to be negative and not positive.

Systems ready to adopt CP as a model of care will require administrative and provider support and financial resources for the start-up of CP. Sustainability of the model over time requires ongoing administrative, provider, system, and financial support. Health care systems will also need to have an adequate volume of prenatal care patients to successfully implement and sustain CP. Furthermore, having an appropriate space to conduct group prenatal appointments, having the providers and other health care professionals trained for group assessments, and having a facilitative leadership style will be necessary. It is possible for CP to improve the quality of traditional prenatal care, the health of pregnant women and their babies, and costs of care, but only if we disrupt the institutions and beliefs in which the status quo of care operates within those institutions [40]. If CP is to become the ubiquitous standard of prenatal care practice, population level improvements in birth outcomes (i.e., effectiveness) may be realized due to a higher quality of care that is respectful and responsive to individual needs (i.e., patient-centered), more timely care, synergistic of resources and energies (i.e., efficiency), and equitable regardless of race, geography, and socioeconomic status (i.e., equitableness).

## Figures and Tables

**Table 1:** Characteristics of survey respondents.

Respondent characteristics							
Variable	*n*	%	Adopters	Nonadopters	*χ* ^2^	df	*P* value
Sex	223						
Male	43	19.5%	20.9%	21.5%	0.037	1	0.848
Female	177	80.5%	79.1%	75.9%			
Race							
White, nonhispanic	142	70.6%	**83.0%**	**47.6%**	**27.264**	**3**	**0.000**
Black, nonhispanic	31	15.4%	**10.4%**	**23.8%**			
Hispanic	6	3.0%	**0.0%**	**9.5%**			
Other	22	10.9%	**6.6%**	**19.0%**			
Age category							
18–24	14	6.3%	**5.6%**	**9.5%**	**20.095**	**3**	**0.000**
25–39	95	44.4%	**59.3%**	**28.4%**			
40–55	71	33.2%	**27.8%**	**37.8%**			
55+	34	15.9%	**7.4%**	**24.3%**			
Do you currently provide care for pregnant women? (yes)	159	76.1%	**86.5%**	**62.5%**	**13.759**	**1**	**0.000**
Profession							
Physician	86	38.9%	43.6%	49.4%	8.837	4	0.065
Nurse practitioner	28	12.7%	9.1%	19.5%			
Certified nurse midwife	1	0.5%	0.9%	0.0%			
Physician's assistant	3	1.4%	0.9%	2.6%			
Other	103	46.6%	45.5%	28.6%			
Your role in the system of care for direct providers							
Patient care	158	72.5%	81.3%	87.0%	4.839	4	0.304
Research	4	1.8%	0.9%	2.6%			
Teaching	16	7.3%	4.7%	0.0%			
Administration	33	15.1%	10.3%	7.8%			
Other	7	3.2%	2.8%	2.6%			
Have you heard of CP? (yes)	206	34.0%	**44.1%**	**9.0%**	**24.188**	**1**	**0.000**
Do you understand the increased value of CP? (yes)	194	91.2%	88.8%	90.5%	0.183	2	0.912
Could you provide a brief explanation of the content and goals of the CP model of care? (yes)	193	91.7%	92.8%	90.5%	0.541	2	0.763

Site characteristics							
Variable	Sites	Respondents %/people/minutes (*n*)	Adopters Sites/%/people/minutes (*n*)	Nonadopters People/minutes (*n*)	*t*	df	*P* value

Number of prenatal care, number of direct services sites	17	100% (223)					
Site services							
Direct	15	84.8% (189)	8 sites (110)	7 sites (79)			
Essential	2	15.2% (34)					
Average weekly patient volume for direct providers*	15	55 people (149)	63 (85)	44 (64)	1.526	147	0.129
Average minutes spent during each patient visit*	15	18.0 minutes (155)	18.8 (92)	18.1 (63)	0.184	152	0.854

*Limited to those who provide direct services.

Statistical significance is highlighted by bold font.

**Table 2:** Essential elements of CP.

	Pretest % (*n*)	Posttest % (*n*)	*t*	df	*P*	Posttest % (*n*)
Have you heard of Centering Pregnancy? (yes)	100% (70)	100% (70)				34.0% (206)
*Essential elements *						
Patients chart their own health (weight, blood pressure, etc.).	60.3% (63)	88.9% (63)	**−4.349**	**62**	**0.000**	83.3%
Health assessments occur in the group space (fundal height, heart rate)	32.3% (62)	71.0% (62)	**−5.231**	**61**	**0.000**	59.8%
Education covers multiple topics and along with support is key difference of CP compared to traditional PNC	87.3% (63)	95.2% (63)	**−**1.523	62	0.133	94.9%
Socialization within group prenatal care enhances support	89.1% (64)	100% (64)	**−2.782**	**63**	**0.007**	97.5%
Group cohesion is important for supporting pregnant women	88.9% (63)	100% (63)	**−2.784**	**62**	**0.007**	94.9%
Patient and provider consistency are important	21.7% (60)	56.7% (60)	**−4.475**	**59**	**0.000**	39.9%
A facilitative leadership style is used	31.2% (61)	67.2% (61)	**−5.133**	**60**	**0.000**	48.9%
Each session has a plan	74.2% (62)	88.8% (62)	**−2.012**	**61**	**0.049**	86.9%
Attention is given to the core content	74.6% (63)	88.9% (63)	**−2.011**	**61**	**0.049**	85.6%
Group conduct honors each member	87.3% (63)	96.8% (63)	**−**1.938	62	0.057	92.4%
The group is conducted in a circle	47.6% (63)	95.2% (63)	**−7.508**	**62**	**0.000**	90.1%
Group size is optimal to promote the process	80.0% (64)	100% (64)	**−4.007**	**63**	**0.000**	95.1%
Involvement of family support people is optional	63.5% (63)	88.9% (63)	**−4.252**	**62**	**0.000**	79.8%
There is ongoing evaluation of outcomes	85.9% (64)	96.9% (64)	−2.420	63	0.018	92.3%
Correct overall percent	**63.5% (55)**	**88.4% (55)**	**−7.052**	**54**	**0.000**	**81.4% (171)**

Statistical significance is highlighted by bold font.

**Table 3:** Patient level indicators: safety, patient centeredness, timeliness, efficiency, and equitableness.

	Overall improve with CP				Adopters improve with CP				Nonadopters improve with CP			
	All	Usual care: high	Usual care: low	*P* value^#^	All	Usual care: high	Usual care: low	*P* value^#^	All	Usual care: high	Usual care: low	*P* value^#^
Safety	35.1%	32.4%	43.2%	0.063	34.0%	29.6%	46.2%	0.311	37.3%	*37.5% *	*36.4% *	∗
Equitableness	**49.0%**	46.5%	65.0%	**0.000**	**52.1%**	49.4%	66.7%	0.000	43.4%	*41.7% *	*60.0% *	∗
Effectiveness	**64.2%**	61.5%	76.9%	**0.000**	**66.7%**	63.3%	82.4%	0.000	59.6%	*58.1% *	*66.7% *	∗
Efficiency	**72.3%**	66.7%	79.7%	**0.000**	**76.8%**	73.1%	81.4%	0.000	**64.2%**	*56.3% *	*76.2% *	0.011
Patient centeredness	**74.3%**	73.2%	77.8%	**0.000**	**80.2%**	80.6%	79.2%	0.000	63.5%	*60.0% *	*75.0% *	∗
Timeliness	**77.0%**	73.3%	80.8%	**0.000**	**77.1%**	70.8%	83.3%	0.000	**76.9%**	*77.8% *	*76.0% *	0.006

^#^McNemar test of significance.

∗: cell count less than 5.

Statistical significance is highlighted by bold font.

**Table 4:** System level differences: RE-AIM.

System characteristics	Usual prenatal care			CP prenatal care			*χ* ^2^	df	*P* value
	Low	Medium	High	Low	Medium	High			
Reach	16.0%	71.2%	12.9%	1.8%	34.1%	64.0%	2.398	4	0.663
Efficacy	13.1%	71.0%	15.0%	0.6%	28.8%	70.6%	3.102	4	0.541
Adoption	21.2%	66.0%	12.8%	11.5%	46.2%	42.3%	**13.045**	**4**	**0.011**
Implementation	7.1%	76.8%	16.1%	1.3%	38.2%	60.5%	5.609	4	0.23
Maintenance	8.2%	67.1%	24.7%	4.4%	35.8%	59.7%	**11.197**	**4**	**0.024**

Statistical significance is highlighted by bold font.

**Table 5:** Strengths and barriers.

	Current prenatal care (*N* = 223)			CP prenatal care (*n* = 223)			*χ* ^2^	df	*P* value
	S	U	B	S	U	B			
Administrative support	**61.1%**	**21.1%**	**17.7%**	**42.9%**	**38.4%**	**18.6%**	**38.68**	**4**	**0.00**
Nurse support	79.7%	13.0%	7.3%	51.4%	32.4%	16.2%	±		
Physician support	82.8%	12.6%	4.6%	54.1%	32.4%	16.2%	±		
Professional group leadership skills*	58.0%	36.2%	5.7%	65.1%	26.3%	8.6%	±		
Client base	62.8%	20.9%	16.3%	57.5%	33.9%	8.6%	±		
Current client satisfaction	60.0%	33.1%	6.9%	62.9%	34.3%	2.9%	±		
Patient flow	47.4%	25.1%	26.9%	59.5%	30.6%	9.8%	±		
PNC education	47.2%	40.9%	11.9%	59.5%	34.5%	6.0%	±		
Language issues**	**40.8%**	**29.3%**	**29.9%**	**35.1%**	**36.8%**	**28.1%**	**51.39**	**4**	**0.00**
Child care	**23.0%**	**32.8%**	**44.3%**	**26.4%**	**31.6%**	**42.0%**	**33.85**	**4**	**0.00**
Transportation	**29.9%**	**28.7%**	**41.4%**	**29.3%**	**31.6%**	**39.1%**	**42.45**	**4**	**0.00**
Parking	55.8%	29.1%	15.1%	50.6%	36.6%	0.128	±		

S: strength; U: uncertain; B: barrier.

±: more than 20% of any cells had less than five, and thus chi-square analysis was unstable and thus not used.

Statistical significance is highlighted by bold font.
